# Choosing a mate in a high predation environment: Female preference in the fiddler crab *Uca terpsichores*

**DOI:** 10.1002/ece3.2510

**Published:** 2016-09-27

**Authors:** Daniela M. Perez, John H. Christy, Patricia R. Y. Backwell

**Affiliations:** ^1^ Research School of Biology The Australian National University Canberra ACT Australia; ^2^ Smithsonian Tropical Research Institute Balboa, Ancón Panama

**Keywords:** courtship, sensory trap, male location, male quality, wave display, drumming display

## Abstract

The interplay between a receiver's sensory system and a sender's courtship signals is fundamental to the operation of sexual selection. Male courtship signals that match a female receiver's preexisting perceptual biases can be favored yet the message they communicate is not always clear. Do they simply beacon the male's location or also indicate his quality? We explored this question in a species of fiddler crab *Uca terpsichores* that courts under elevated predation risk and that mates and breeds underground in the safety of males' burrows. Sexually receptive females leave their own burrows and are thereby exposed to avian predators as they sequentially approach several courting males before they choose one. Males court by waving their single greatly enlarge claw and sometimes by building a sand hood next to their burrow entrance. Hoods are attractive because they elicit a risk‐reducing orientation behavior in females, and it has been suggested that claw waving may also serve primarily to orient the female to the male. If the wave communicates male quality, then females should discriminate mates on the basis of variation in elements of the wave, as has been shown for other fiddler crabs. Alternatively, variation in elements of the claw waving display may have little effect on the display's utility as a beacon of the location of the male and his burrow. We filmed courting males and females under natural conditions as females responded to claw waving and chose mates. Analysis of the fine‐scale courtship elements between the males that females rejected and those they chose revealed no differences. When predation risk during courtship is high, males' courtship displays may serve primarily to guide females to safe mating and breeding sites and not as indicators of male quality apart from their roles as beacons.

## 1 Introduction

Preexisting biases in female sensory systems can play an important role in the evolution of mating signals (Ryan & Cummings, 2013). Mate‐attraction signals can mimic triggers of positive responses in females; for example, orange coloration in guppies that eat orange food items (Rodd, Hughes, Grether, & Baril, 2002; but see Fuller, Houle, & Travis, 2005). Male signals, however, can also evolve to exploit negative responses in females, such as predator‐avoidance responses (Pascoal, Moran, & Bailey, 2016). In a lebinthine cricket, the male mate‐attraction signal is very similar to the high frequency calls of their bat predators (ter Hofstede, Schoneich, Robillard, & Hedwig, 2015) and females show a startle response on hearing the male signal: they vibrate their legs which moves the leaf they are standing on, allowing the male to locate them (ter Hofstede et al., 2015). This phenomenon is called a “sensory trap” as the signaller exploits a stimulus–response relationship of the receiver that usually functions in another context; in this case, predator detection (Christy, 1995).

**Figure 1:**
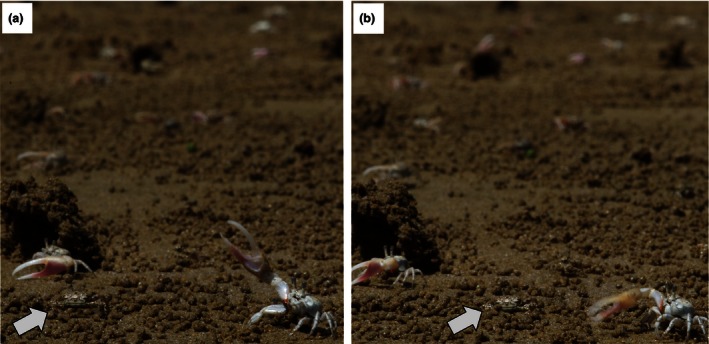
Female of *Uca terpsichores* indicated by the arrows in the bottom‐left (a) and center (b) watching the courtship displays of the male on her right displaying (a) waves and (b) drummings. Edited and printed with permission of Dr. Tanya Detto

When mating signals originate as sensory traps, they stimulate a sensory bias for perception of other stimuli (Fleishman, 1988; Guilford & Dawkins, 1991; Scheffer, Uetz, & Stratton, 1996). The courtship display of fiddler crabs (*Uca* spp., Crustacea: Ocypodidae; Figure 1) is thought to have originated in this way. The predator‐detection response of females appears to form the basis of a sensory trap (Burford, McGregor, & Oliveira, 2000; Christy, 1995; Oliveira & Custodio, 1998). During displays, male fiddler crabs wave and extend their large claws above the female's visual horizon (Crane, 1975; How, Zeil, & Hemmi, 2009). In their flat, two‐dimensional world, the visual system is adapted to categorizing objects above a crab's visual horizon as potential predators (Land & Layne, 1995; Zeil & Hemmi, 2006; Zeil, Nalbach, & Nalbach, 1986). When the claw is raised above the female's visual horizon, it attracts her attention as do moving predators (Zeil & Al‐Mutairi, 1996).

Signals that capture the female's attention also reveal the male's location and identity (Reichert, 2015; Stamps & Barlow, 1973) and the location of his burrow (Christy & Salmon, 1991) where the female may shelter temporarily from predators (Peso, Curran & Backwell, 2016; Ribeiro, Christy, Rissanen, & Kim, 2006). Males increase their wave rate when they detect wandering females, and this further increases their conspicuousness and locatability (Milner, Jennions, & Backwell, 2010; Sanches & Backwell, In Prep.).

Further evolution of such signals may facilitate female assessment of male quality (Ryan & Rand, 1993). *Uca perplexa* males modulate their wave structure as the female gets nearer: the long‐range signal is more visible as it has a larger sweep; the close‐range signal is more rapid as it has a smaller sweep and is thought to contain information about male quality (How, Hemmi, Zeil, & Peters, 2008). In this species, females select males with more rapid waves and males whose waves have longer upstroke durations (Murai & Backwell, 2006). Other studies showed that females of *U. crenulata*,* U. tangeri*, and *U. perplexa* select higher waves (de Rivera, 2005; Murai & Backwell, 2006; Oliveira & Custodio, 1998). Another well‐documented female preference in fiddler crabs is for males that wave slightly before (give “leading waves”) their neighbors in a group of males waving nearly synchronously (Backwell, Jennions, Passmore, & Christy, 1999).

In studies of female choice of male quality, it is easy to assign the wrong function to a signal (Ryan & Cummings, 2005). Signal features that may act as a handicap or signal male quality are often the same ones that increase conspicuousness or locatability of a signaller (Mowles & Ord, 2012). Rapid waving, higher waves, and leading waves are expensive to produce and are likely candidates for mate choice of male quality (Mowles, 2014). They are also all likely to increase male detectability and locatability (Ryan & Cummings, 2005). One way to examine the use of mating displays as signals of male location or quality is to examine species with high levels of predation. Differential male investment in courtship would indicate whether, under high predation risks, the signal is a handicap. However, when the cost of searching for a mate is elevated due to predation risk, female fiddler crabs should quickly and accurately detect and locate potential mates and their burrows into which they can retreat for safety and spend little time in risky assessment of traits only indicative of male quality. Low levels of selection on male traits that do not decrease the risk of searching for a mate may result in a decreased use of the male's signals of his quality.

Predation on adult fiddler crabs is much higher in the Americas than in old‐world species (Backwell et al., 1998; Christy, 2007; Ribeiro et al., 2006). It is therefore not surprising that most structure‐building fiddler crabs are in an American clade (Christy, 2007): vertical mud pillars (*U. beebei*) or hoods (*U. terpsichores*) next to a male's burrow entrance have been shown to guide the female to the male's burrow (Christy, 1988; Christy, Backwell, Goshima, & Kreuter, 2002; Christy & Salmon, 1991; Ribeiro et al., 2006) as does the raised carpus display of *U. beebei* (Christy, 1988), which exaggerates the movement males make when they enter their burrows. The attractiveness of both pillars and hoods to females increases with perceived predation risk (Kim, Christy, & Choe, 2007; Kim, Christy, Dennenmoser, & Choe, 2009). Seismic signals are also thought to attract the female's attention and beacon his location (Christy & Salmon,^1991^; Figure 1b) although once a female is at the male's burrow, they also may signal male size or stamina (Takeshita & Murai, 2016).

In species with high levels of predation, such as *U. terpsichores* (Christy, 2007), we would expect the male wave display and seismic drumming to function primarily in male detection and localization. As it is more costly (time and risk) for females to compare males and make the fine‐scaled choices we see in the old‐world species, we expect variation in features of the wave display and seismic drumming to play a lesser role in mate choice (Christy, 2007). Here, we examine the differences in waving and drumming between a male that was chosen by a female and his nearest, nonselected, neighbor. By video recording natural female approaches to courting males, we were able to compare the two males for the number of waves and drums given, the structure of their waves and drums, their size, their ability to produce leading waves, and the possession of a sand hood.

## 2 Methods

### 2.1 Species and study site

We studied a population of *Uca terpsichores* on the mudflats of the Base Naval Vasco Nuñez de Balboa, Panama (8°56′55″N; 79°34′25″W) in December 2013–January 2014. *U. terpsichores* lives in mixed sex, high‐density populations on sand flats and bars in Central American bays and estuaries and are extensively predated by great‐tailed grackles, *Quiscalus mexicanus* (Kim et al., 2007; Koga, Backwell, Christy, Murai, & Kasuya, 2001; Koga, Backwell, Jennions, & Christy, 1998). The courtship wave is circular–lateral in form, starting with a frontal extension with the claw followed by a gradual rise above the eyestalks and finishing with a faster downward more vertical movement (Figure 1a). Seismic signals are also common in this species (Figure 1b) and consist of bouts of fast low amplitude vertical waving of the claw directly in front of the body, with the claw striking the substrate and producing vibrations (Müller, 1989). We did not directly measure the seismic component of this sharp vertical wave in this study. When a female fiddler crab of a species that mates in males' burrows is ready to mate, she leaves her territory and wanders through the population of courting males (Christy, 1983; Christy & Salmon, 1991; Christy et al., 2002; de Rivera, 2005). Male courtship encourages females to approach their burrows, and she moves from one burrow to the next, visiting a succession of males. By briefly entering the burrow, the female is probably able to assess its quality as it has been shown in many fiddler crab species that females are very selective on burrow features (Backwell & Passmore, 1996; Christy, 1983; Reaney & Backwell, 2007). Courtship at the burrow entrance can also take place as vibrations produced by rubbing the ridged lower inner surface of their claws against tubercles on their first walking legs (Müller, 1989; superficially). We did not measure this stridulatory behavior or burrow quality because we were interested only in signals of competing males that females could assess before they reached their burrows: the signals that attract females to males and their burrows.

### 2.2 Video recording

We filmed naturally mate‐searching females as they wandered through the population of courting males. Mate‐searching females are easily found by looking for small clusters of fast‐waving males facing toward the female in the middle of the cluster. Once we located a wandering female, we watched her until she had visited and left a male (so that we were sure she was mate searching), and then we filmed her using a JVC GZ‐EX355BAA video camera as she approached the next male. We used a close frame that included the female and the small set of males in her vicinity (±2–4 males; 50 cm frame). The camera was supported on a tripod 50 cm high, so the camera‐to‐male angle did not differ by more than 2° between videos. After filming, we placed a vertical and horizontal scale in the frame and filmed it, allowing us to adjust for the angle between the camera and crab. This facilitated measurements of the crab size and the wave features. The definition and clarity were sufficient to allow for very accurate measurements from the videos. We excluded all videos in which the crabs were startled by predators, the males fought each other, the female failed to make a choice, or any of the crabs showed a scare response (run to and enter their burrows or freeze). We made complete recordings of 28 unique female visits (no crab appeared in more than one of the videos).

### 2.3 Digitalization

We digitalized the videos using a frame‐by‐frame analysis at 30 frames per second using the software digilite created in MATLAB (The MathWorks, Inc., Natick, MA, USA) by Jan Hemmi and Robert Parker of The Australian National University. The software enables pinpointing of “landmarks” on the males with enlarged claw and body.

We used the scale in each video sequence to measure (1) the claw length of the visited male and (2) the claw length of the nearest male to the visited male (i.e., the nearest nonselected male that courted the female). Each mate choice sequence was split into two periods: (1) the period during which the female stopped moving and watched the courting males (hereafter called “before choice”) and (2) the period during which the female moved toward the selected male (hereafter called “during choice”). We considered that the female made her choice once she reached the selected male's burrow, and he ceased waving or drumming (we used the same cutoff point for both the selected and nonselected male). We counted the number of waves and/or drums given by the selected and nonselected male during the two mate choice periods. For every wave and drum of both males during both periods, we measured the distance the claw tip moved in the upswing and the downswing of the wave/drum (hereafter called the “up distance” and “down distance”). We also measured the duration of each of these movements (“up duration” and “down duration”). For each wave, we measured the maximum height that the claw tip reached (relative to the height of the eyestalk base). We noted whether the male had a sand hood at his burrow entrance. Finally, we examined wave timing between the selected and nonselected males by first identifying which of their waves overlapped and then noting which of the males produced the leading wave (the first wave to start in an overlapping wave pair).

### 2.4 Analysis

We compared the selected and nonselected males using a mixed model but found that the residuals were not normally distributed. Data transformations did not normalize the residuals, so we used nonparametric tests throughout. We used Wilcoxon tests to compare the (1) number of waves or (2) drums given by the selected and nonselected males and (3) the claw lengths of the selected and nonselected males. We tested whether selected or nonselected males differed in their likelihood of having a sand hood at their burrow entrance using a Fisher's exact test. We compared the number of leading waves produced by selected and nonselected males running a Wilcoxon test.

To accommodate the high number of “wave/drum structure” variables measured, we ran a principal component analysis (separately on the waves and drums). We used the mean value for each wave/drum variable (up distance, up duration, down distance, down duration, and maximum height for waves only) for each male. We then used the PC scores to compare the selected and nonselected males (waves and drums analyzed separately) using Wilcoxon tests.

Statistics were conducted in SPSS 20 and we set α = .05. We calculated the effect size of the Wilcoxon tests (*r* = *Z*/√*n*).

## 3 Results

Selected and nonselected males did not differ in size (selected male claw length $\overset{¯}{x}$(*SD*) = 1.49 (0.15) cm, *n* = 28; nonselected male claw length $\overset{¯}{x}$(*SD*) = 1.52 (0.20) cm, *n* = 28; Wilcoxon test *Z* = −0.48, *p* = .63). They also did not differ in the number of drums they produced, either before or during the process of choice (Table 1). Before the female started moving to the selected male, both males produced the same number of waves (Table 1); but while the female was moving toward the male, the selected male produced fewer waves than the nonselected male (Table 1). This result is robust, even with a false discovery rate test (Table 1).

**Table 1:** Mean, *SD* and number of waves, and drums produced before and during female choice

	Before		During	
	Wave	Drum	Wave	Drum
Selected				
Mean	4.74	9.95	0.33	2.17
*SD*	4.52	7.66	0.56	3.81
*n*	19	19	24	24
Nonselected				
Mean	4.63	4.11	0.63	1.00
*SD*	5.43	14.74	0.77	1.74
*n*	19	19	24	24
Wilcoxon				
*Z*	−0.11	−1.01	−2.11	−1.53
*p*	.91	.31	.04	.13
FDR				
*p<(i*/*m) Q*	NS	NS	SIG	NS

The comparison of number of displays given selected and nonselected males is tested by a Wilcoxon test *Z*.

The structure of the wave (the distance and duration of claw movement during waves and drums and the maximum wave height) also did not differ between the selected and nonselected males. The weighting of each measured wave/drum variable on the principle component score is given in Table 2, and the comparisons between selected and nonselected males are given in Table 3.

**Table 2:** The proportion of variance explained by each component of the three principle components (two wave PCs and one drum PC) from the PCA of wave and drum structure

	Weightings	
	PC1	PC2
Wave		
Up distance	0.94	0.04
Down distance	0.90	0.20
Up duration	0.90	−0.09
Down duration	0.14	−0.70
Mean height	0.01	0.73
Max height	0.93	0.05
Drum		
Up distance	0.92	
Down distance	0.95	
Up duration	0.73	
Down duration	0.38	

**Table 3:** The mean, standard deviation, and sample size of selected and nonselected males for each trait: the number of waves given; the number of drums given; male claw size; the two wave principle components; and the one drum principle component

	Mean (*SD*)	*n*	Wilcoxon	
			*Z*	*p*
No. of waves				
Selected	4.12 (2.78)	17	−1.07	.29
Nonselected	6.89 (5.78)	18		
No. of drums				
Selected	11.96 (11.80)	25	−1.03	.30
Nonselected	15.57 (13.84)	21		
Size				
Selected	1.49 (0.15)	26	−0.48	.63
Nonselected	1.52 (0.20)	26		
Waves PC1				
Selected	−0.57 (1.16)	18	−0.04	.97
Nonselected	0.05 (0.86)	20		
Waves PC2				
Selected	0.20 (0.96)	18	−0.31	.75
Nonselected	−0.18 (1.01)	20		
Drums PC1				
Selected	−0.20 (1.03)	25	−1.79	.07
Nonselected	0.24 (0.92)	21		

The comparisons between selected and nonselected males are given as the test statistic (*Z*) from Wilcoxon tests, the *p* value associated with the Wilcoxon *Z*. The sample sizes differ between selected and nonselected males because not all males produced waves or drums. The sample sizes for data used in the Wilcoxon tests are the lower value out of the selected and nonselected males.

Selected and nonselected males did not differ in whether or not they had sand hoods next to their burrows (although the sample size was too small to make inferences: 6/29 selected males had hoods, 2/29 nonselected males had hoods, Fisher's exact test *p* = .25).

Less than half of the males produced waves that overlapped with those of their neighbors (11/26). There was a total of 42 waves that overlapped between the selected and nonselected males. In those cases where waves overlapped, 10/11 of the nonselected males produced the leading wave (binomial *p* = .01, *n* = 11). When considering all the overlapping waves, the nonselected males were more likely to produce the leading wave (selected males produced 15 leading waves, nonselected males produced 27 leading waves; Wilcoxon test *Z* = −2.09, *p* = .04).

## 4 Discussion

Males that were selected by a female were not different to their nonselected neighbors in any of the measured wave or drum variables or in the size of the males. Selected and nonselected males were indistinguishable except for two differences: selected males produced fewer waves than nonselected males while the female was moving toward the males, and selected males were less likely to produce leading waves than were nonselected males. As we were able to detect these differences, we are confident that the negative results we found for the other variables are not due to sample sizes.

Claw waving in fiddler crabs is often thought to be a model example of a display selected by female choice (Christy, 2007), and there are many studies of old‐world fiddler crabs that have shown differences between a selected male and his immediate neighbors: In *Uca annulipes*, the selected male had a higher wave rate and was more likely to produce leading waves than was his neighbor (Backwell et al., 1999). In *U. mjoebergi*, selected males waved at a higher rate than their neighbors (Callander, Kahn, Maricic, Jennions, & Backwell, 2013). In *U. perplexa*, the wave structure of selected males was different to nonselected males: The claw was raised higher, the claw was lowered later and to a higher resting position than in nonselected males (Murai & Backwell, 2006).

Why, in *U. terpsichores*, do selected and nonselected males not differ in any of the wave features we measured and that are used by other species of fiddler crabs to choose mates? It is possible that females use other male traits that we did not measure to choose mates. We think this is unlikely, however, because selected traits in fiddler crabs usually are highly correlated (Christy et al., 2002; Cummings, Jordão, Cronin, & Oliveira, 2008; de Rivera, 2005; Jennions & Backwell, 1998; Murai & Backwell, 2006; Oliveira & Custodio, 1998). Fiddler crab females could benefit from mainly assessing display timing and amplitude of waves as well as seismic signals as these traits can be perceived from any direction that the female approaches the male. Female assessment of other male traits would be restricted to specific signaller–receiver orientations, such as in *Hypolimnas bolina* butterflies where signal effectiveness relies on its spatial direction (White, Zeil, & Kemp, 2015).

A more likely explanation for the similarity between the signals of selected and nonselected males is that females do not choose mates based on variation in these traits. In many of the fiddler crab species that appear to have mate choice based on wave traits, there are low levels of predation (Backwell pers. obs. for *U. annulipes, U. mjoebergi, U. perplexa*; de Rivera, 2005 for *U. crenulata*). For the study species here, however, the great‐tailed grackle, *Quiscalus mexicanus*, is a common and persistent avian predator (Kim et al., 2007; Koga et al., 1998). The high levels of risk may prevent females of this species from finely comparing male and wave traits. Fiddler crab females from Indo‐Pacific species in fact take more time to walk between burrows during mate searching (Backwell pers. obs.) evidencing the effects of high predation on mate preference in New World species (Christy, 2007). Thus, females could still allow enough time between moving away from the last visited male to the next to judge male courtship.

Selection by predation for rapid assessment of male traits may not be the only explanation for the similarity in waving and drumming between chosen and rejected males. Males are also under high predation risk and vigorous courtship could cost their lives (Koga et al., 2001). Predators are more likely to spot a male fiddler crab that possess an enlarged claw and is usually more brightly colored than females (Cummings et al., 2008; Koga et al., 2001). In brush‐legged wolf spiders, courting males are significantly more predated by frogs when displaying pronounced decorative traits (Clark, Zeeff, Karson, Roberts, & Uetz, 2016). The fringe‐lipped bat is more attracted by the multicomponent sexual signal from túngara frogs, call and song sac movement, than by the male's advertisement call alone (Halfwerk et al., 2014). Conspicuous signals could be selected as an indication of honesty through the handicap principle (Mowles & Ord, 2012; Zahavi, 1975) as higher courtship efforts result in elevated predation costs for signallers (Roberts, Taylor, & Uetz, 2007). However, preferences on fine scale of the sexual signals can be reduced when females are also under high predation threat (Breden & Stoner, 1987). This should weaken selection on *U. terpsichores* males to court more vigorously than their immediate neighbors. Courtship effort can indeed significantly decrease under intense predation, as has been shown experimentally (Koga et al., 1998).

If females are not assessing males on the basis of size, visual, or seismic signals and are concerned only with the rapidly detecting and locating a male, why do they visit and reject several males before selecting a mate? The sampling behavior may be due to the females searching for a burrow suitable for incubation. Burrow quality has been consistently found to be important in fiddler crabs (Backwell & Passmore, 1996; Christy, 1983, 1988; de Rivera, 2005; Reaney & Backwell, 2007). In most species, it appears that the female's final decision to mate is based on the direct assessment of burrow structure (Backwell & Passmore, 1996; de Rivera, 2005). In *U. terpsichores*, the waving and drumming displays may allow the female to detect and quickly locate the male so that she can assess the structure of the male's burrow, rather than as signals by which females choose mates.

The fiddler crab waving display may attract the attention of females because it includes rapid, elevated motions of the claw that are seen in the portion of the visual field that crabs use to detect predators and where their resolution of vertically moving objects is sharpest (Burford et al., 2000; Christy, 1995; Christy, Backwell, & Schober, 2003; Christy, Baum, & Backwell, 2003; Oliveira & Custodio, 1998; Zeil et al., 1986). Hence, male claw waving may stimulate a visual bias established by selection for predator detection. Indeed, two rare displays of male *U. terpsichores*, not recorded in this study, elicit predator avoidance responses in females; both include rapid elevated movements of males or their claws (Christy, 2007). Clearly, however, females are attracted to not startled away from typical male claw waving and drumming displays. In this manner, a sensory bias selected for predator detection can be incorporated into courtship allowing females to rapidly locate the signaller to their benefit under high predation risk (Andersson, 1982; Arnqvist, 2006; Dawkins & Guilford, 1996; How et al., 2008). The ability of females to detect and locate courting males and their burrows is at a selective premium in the heavily predated Central American fiddler crabs: *U. terpsichores* males build sand hoods at their burrow entrances and females orient to hoods to find males' burrows (Christy et al., 2002; Kim et al., 2007). In *U. beebei*, the mud pillar males construct at the entrance to their burrows function in the same way (Christy, 1995; Christy, Backwell, et al., 2003;. Kim et al., 2009). The dark inner carpus of the claw of male *U. beebei*, an unusual character in the genus, is displayed to the female as the male enters his burrow, and though experimental evidence is lacking, is thought to visually guide an approaching female to the male's burrow (Christy, 1988). In both these species, the high predation levels are likely to have driven selection on males to facilitate the rapid detection and locatability of their burrows to wandering females (Christy, Backwell, et al., 2003; Christy, Baum, et al., 2003; Kim et al., 2007, 2009). Although we did not find a preference for males that had hoods, our sample size was too small to detect it (six of the selected males had hoods; two of the nonselected males had hoods). The stimulation of sensory biases by courtship signals does not imply, and there is no role for female choice (Fuller et al., 2005). It is possible, but not necessary, that the wave is condition‐dependent and could therefore also mediate female choice of good condition males (Christy,^1995^; Ryan & Rand, 1993).

As a female moves away from the last visited burrow, she could be attracted to the next nearest male as signals are stronger at shorter distances. However, females often pass closer males to visit others further away (authors pers. obs.). This behavior is puzzling as we found no preferences on male size or signals. We know that the presence of a hood makes a male more attractive and that the hood itself partially explains this (Christy et al., 2002). However, we do not know why females are orienting to more distant males based on their signals. The hectic and fast runs between males' burrows could themselves explain a randomness of female approach. However, there could also be a more elaborate explanation such as an interaction between the proximity of the courting male to the female and seismic and visual signals he gives. Sound production from seismic signals could account for a differential perception of mate candidates. Although seismic signal assessment is only accurately made at short distance after female approach to the male's burrow (Takeshita & Murai, 2016). In addition, females may be plotting out their next movement based on watching males approach, retreat back to, and possibly enter their burrows while the female is standing away but still safe near the last male's burrow. The apparent exaggeration by the raised carpus display of body movements the male *U*. *beebei* make when they enter their burrows suggests such indicators of burrow location may be important. We did not measure male movements that might reveal the location of their burrows in this study.

### 4.1 Wave leadership and the number of waves

We found two differences between the selected and nonselected males. The selected and nonselected males produced the same number of waves before the female started moving toward them, but the selected male produced fewer waves than the nonselected male once the female was making her approach. This is due to a change in the behavior of the selected male as the female nears him: He runs back to his burrow entrance, leading the female and often darting back‐and‐forth to guide the female. This prevents him from waving at the same rate as the nonselected male. In addition, at this stage, females had already made a choice of which male to approach and the candidate displays would be no longer under evaluation.

The other significant difference we found was in wave leadership: Nonselected males were more likely to produce leading waves. In many of the synchronous waving fiddler crab species, synchrony occurs because adjacent males compete with each other to produce leading waves (Backwell et al., 1998, 1999). Females have a preference for leading waves (Kahn, Holman, & Backwell, 2014). Male *U. terpsichores* do not wave synchronously. Less than half of the males produced waves that overlapped with those of their neighbor. Of the overlapping waves, however, the majority were produced by the nonselected males. This may be a statistical anomaly, but it certainly requires further investigation.

## 5 Conclusion

The variety of wave patterns found among fiddler crab species is still puzzling. Could sexual selection be a strong influence in the evolution of wave display diversity? This study revealed that under high predation risks, female preference for sexual signals are likely due to their role as beacons rather than indicators of male quality. This raises doubt about the power of sexual selection to shape such diversity. However, this study points to a possible origin of the sexual display in stimuli that play to sensory biases selected by predation and studies on other species exposed to a range of predation risk during courtship may reveal how selection shapes the evolution of species‐specific wave displays.

## Conflict of Interest

None declared.
